# Steep rise in drug use-associated infective endocarditis in West Virginia: Characteristics and healthcare utilization

**DOI:** 10.1371/journal.pone.0271510

**Published:** 2022-07-15

**Authors:** Ruchi Bhandari, Talia Alexander, Frank H. Annie, Umar Kaleem, Affan Irfan, Sudarshan Balla, R. Constance Wiener, Chris Cook, Aravinda Nanjundappa, Mark Bates, Ellen Thompson, Gordon S. Smith, Judith Feinberg, Melanie A. Fisher

**Affiliations:** 1 School of Public Health, West Virginia University, Morgantown, West Virginia, United States of America; 2 Health Education and Research Institute, Charleston Area Medical Center, Charleston, West Virginia, United States of America; 3 Joan C. Edwards School of Medicine, Marshall University, Huntington, West Virginia, United States of America; 4 School of Medicine, West Virginia University, Morgantown, West Virginia, United States of America; 5 School of Dentistry, West Virginia University, Morgantown, West Virginia, United States of America; 6 Department of Cardiovascular Medicine, Charleston Area Medical Center, Charleston, West Virginia, United States of America; University of Bologna, ITALY

## Abstract

**Introduction:**

Life-threatening infections such as infective endocarditis (IE) are increasing simultaneously with the injection drug use epidemic in West Virginia (WV). We utilized a newly developed, statewide database to describe epidemiologic characteristics and healthcare utilization among patients with (DU-IE) and without (non-DU-IE) drug use-associated IE in WV over five years.

**Materials and methods:**

This retrospective, observational study, incorporating manual review of electronic medical records, included all patients aged 18–90 years who had their first admission for IE in any of the four university-affiliated referral hospitals in WV during 2014–2018. IE was identified using ICD-10-CM codes and confirmed by chart review. Demographics, clinical characteristics, and healthcare utilization were compared between patients with DU-IE and non-DU-IE using Chi-square/Fisher’s exact test or Wilcoxon rank sum test. Multivariable logistic regression analysis was conducted with discharge against medical advice/in-hospital mortality vs. discharge alive as the outcome variable and drug use as the predictor variable.

**Results:**

Overall 780 unique patients had confirmed first IE admission, with a six-fold increase during study period (p = .004). Most patients (70.9%) had used drugs before hospital admission, primarily by injection. Compared to patients with non-DU-IE, patients with DU-IE were significantly younger (median age: 33.9 vs. 64.1 years; p < .001); were hospitalized longer (median: 25.5 vs. 15 days; p < .001); had a higher proportion of methicillin-resistant *Staphylococcus aureus* (MRSA) isolates (42.7% vs. 29.9%; p < .001), psychiatric disorders (51.2% vs. 17.3%; p < .001), cardiac surgeries (42.9% vs. 26.6%; p < .001), and discharges against medical advice (19.9% vs. 1.4%; p < .001). Multivariable regression analysis showed drug use was an independent predictor of the combined outcome of discharge against medical advice/in-hospital mortality (OR: 2.99; 95% CI: 1.67–5.64).

**Discussion and conclusion:**

This multisite study reveals a 681% increase in IE admissions in WV over five years primarily attributable to injection drug use, underscoring the urgent need for both prevention efforts and specialized strategies to improve outcomes.

## Introduction

Infective endocarditis (IE) is a devastating disease that has been associated with injection drug use [1, 2], resulting in a persistent increase in IE hospitalizations across the U.S. since 2000 [3, 4]. Drug use-associated infective endocarditis (DU-IE) hospitalizations increased by twelve-fold in North Carolina between 2007 and 2017 [5] and eight-fold in Oregon between 2008 and 2018 [6], with the increase most notable in rural areas [7].

West Virginia (WV), the epicenter of the Appalachian opioid epidemic in the U.S., is experiencing multiple injection drug use syndemics, including the highest rates of fatal overdose, acute hepatitis B and neonatal abstinence syndrome in the U.S., as well as unprecedented increases in cases of acute hepatitis C, HIV, and life-threatening bacterial infections such as IE [8, 9]. A study based in one of WV’s largest tertiary hospitals reported that IE admissions increased by 154% between 2012 and 2018 [9]. The injection drug use and IE syndemic represents a burgeoning public health crisis in WV and beyond. As IE has been shown to be a sentinel marker for the injection drug use epidemic [2] and given that WV has the highest fatal drug overdose rate in the nation, we sought to examine trends in IE in the state.

IE results from a bacterial or fungal bloodstream infection that affects the heart valves. In DU-IE, the use of unsterile or shared syringes, contaminated drugs or other equipment, injection behaviors such as licking the needle, and injection through unclean skin facilitates pathogen entry [10]. The resulting transient bacteremia or fungemia infects the endothelial tissue covering cardiac valves that creates a nidus of chronic infection (vegetation) and develops in persistent, high-grade bloodstream infection. Symptoms of IE initially resemble those of other serious bacterial infections with patients commonly presenting with fever, fatigue, weakness, and shortness of breath, along with a new or worsening heart murmur. However, since initial symptoms may be minimal or mimic other conditions, IE can be overlooked, misdiagnosed, and improperly treated. Further, long-term intensive antibiotic therapy and complications of IE, such as the need for surgical intervention and untreated opioid use disorder, make DU-IE challenging and expensive to treat. Moreover, DU-IE carries higher rates of comorbidity and readmissions for recurrent infections compared to non DU-IE [3, 4]. Inherent nonadherence in this population exacerbates the need for care coordination, particularly medication for opioid use disorder (MOUD). Studies have shown the effectiveness of MOUD in improving patient outcomes, such as treatment completion and reducing readmissions [11].

A critical barrier to advancing DU-IE treatment and prevention is the limited information available on patients with DU-IE, including substance use behaviors, clinical characteristics, and healthcare utilization, especially in studies relying just on hospital discharge data and ICD codes [5–7]. To fill this gap, we created a multisite database of confirmed IE cases admitted at four university-affiliated referral hospitals in WV with advanced cardiac care capability, three of which perform all the cardiac surgeries in the state. The objective of our study is to utilize this statewide database to describe the epidemiology, characteristics related to substance use behavior, and healthcare utilization among DU-IE and non-DU-IE patients over the five-year period from January 2014 to December 2018, during which overdose rates increased dramatically. We explore key metrics, such as continuation of MOUD during hospitalization, that impact hospital stays and readmission rates. Lastly, we examined the factors that independently predict treatment completion among this population. We sought to determine variables associated with better outcomes, which may be used to inform strategies for specialized treatment to improve outcomes.

## Materials and methods

### Study design and inclusion/exclusion criteria

This study was a multicenter retrospective electronic medical records (EMR) review of adults 18 to 90 years of age who had their first hospital admission for IE between January 1, 2014 and December 31, 2018 at any of the four university-affiliated referral hospitals with advanced cardiac care capability in WV. As IE is not a reportable condition, there are no published data for the number of IE cases at each healthcare facility in WV; however, state experts in infectious diseases and cardiology estimate that given the emergent need for sophisticated cardiac and/or surgical intervention, most of the IE cases statewide are transferred to the four facilities.

Patients were initially identified using the International Classification of Diseases, Tenth Revision, Clinical Modification (ICD-10-CM) codes for IE (B376, I33.0, I33.9, I34.0, I34.8, I35.0, I35.1, I35.2, I35.8, I35.9, I36.0, I36.8, I37.0, I37.8, I38, I39), followed by a detailed manual review of the electronic medical records (EMR) of all admissions to confirm diagnosis of IE. We used the General Equivalence Mappings from the Centers for Medicare and Medicaid Services to convert ICD-10-CM to ICD-9-CM codes from previous years [12]. The study was approved by the Institutional Review Board at West Virginia University (protocol 1811373348).

### Data abstraction

EMR data collected from each of the four hospitals were entered into a single, standardized, coded dataset using the Research Electronic Data Capture (REDCap) system [13]. REDCap is a secure, HIPAA-compliant, web-based data capture system hosted at West Virginia University. Data abstractors for each of the hospitals were trained in the use of the local EMR, REDCap, and the variables of interest. The principal investigator (RB) provided detailed decision tree/stem logic to determine if cases met IE criteria for data abstraction and was available to resolve questions in a timely manner. After data for 10 patients from each site were entered into REDCap, we conducted a pilot test to identify errors and omissions, and then modified and clarified variables before proceeding with data abstraction on all potential cases identified by ICD codes.

### Data sources

We abstracted individual patient information from history and physical examination notes, provider notes, consultation notes, hospital narratives, laboratory tests and imaging results, operative notes, and discharge summaries for each patient. Variables were as follows:

- *Demographics*: sex (male, female) and age (continuous and three age-groups:18–44; 45–64; >64 years); race (dichotomized as White vs. others since WV population is predominantly White (93.5%)).
- *Substance use*: smoking status (current smoker, ex-smoker, non-smoker); alcohol use (current alcohol use, prior alcohol use, no alcohol use); drug use (defined as drug use any time before hospital admission), type of drug, method of drug use, participation in MOUD before, during, and at discharge from hospital. Categories for method of drug use (injection, inhalation, ingestion) were not mutually exclusive.
- *Clinical characteristics*: causative organisms identified and co-morbid medical conditions.
- *Hospital resource utilization*: consultations received; surgery for IE (valve replacement, pacing lead, or root replacement); length of hospital stay; discharge status (including discharges against medical advice [AMA]); discharge location; readmission during the study period; and death.

### Statistical analyses

Only the first admission of each unique patient during the study period was analyzed. The number of subsequent admissions was recorded to obtain data on the frequency of readmissions during the study period. Categorical variables are presented as counts and percentages. DU-IE and non-DU-IE groups were compared using Chi-square test or Fisher’s exact test when expected cell count was <5. Continuous variables are presented as median and interquartile range (IQR); medians were compared using Wilcoxon rank-sum test. To examine the relationship of drug use and the combined endpoint of discharge AMA/in-hospital mortality, crude and multivariable logistic regression analyses were conducted with discharge against medical advice/in-hospital mortality vs. discharge alive as the outcome variable and drug use as the predictor variable. Discharge AMA and in-hospital mortality were combined due to the small number of AMA discharges among non-DU-IE and the fact that both are negative outcomes compared to referent category of discharged home. Multivariable analysis was adjusted for sex (male, female), age (18 to <65, 65–90 years), number of comorbidities (0, 1–2, ≥3), and surgery (yes, no). All statistical analyses were conducted in R version 4.0.2 (R Foundation for Statistical Computing). The significance level was set at *p* < 0.05.

## Results

**Fig 1:**
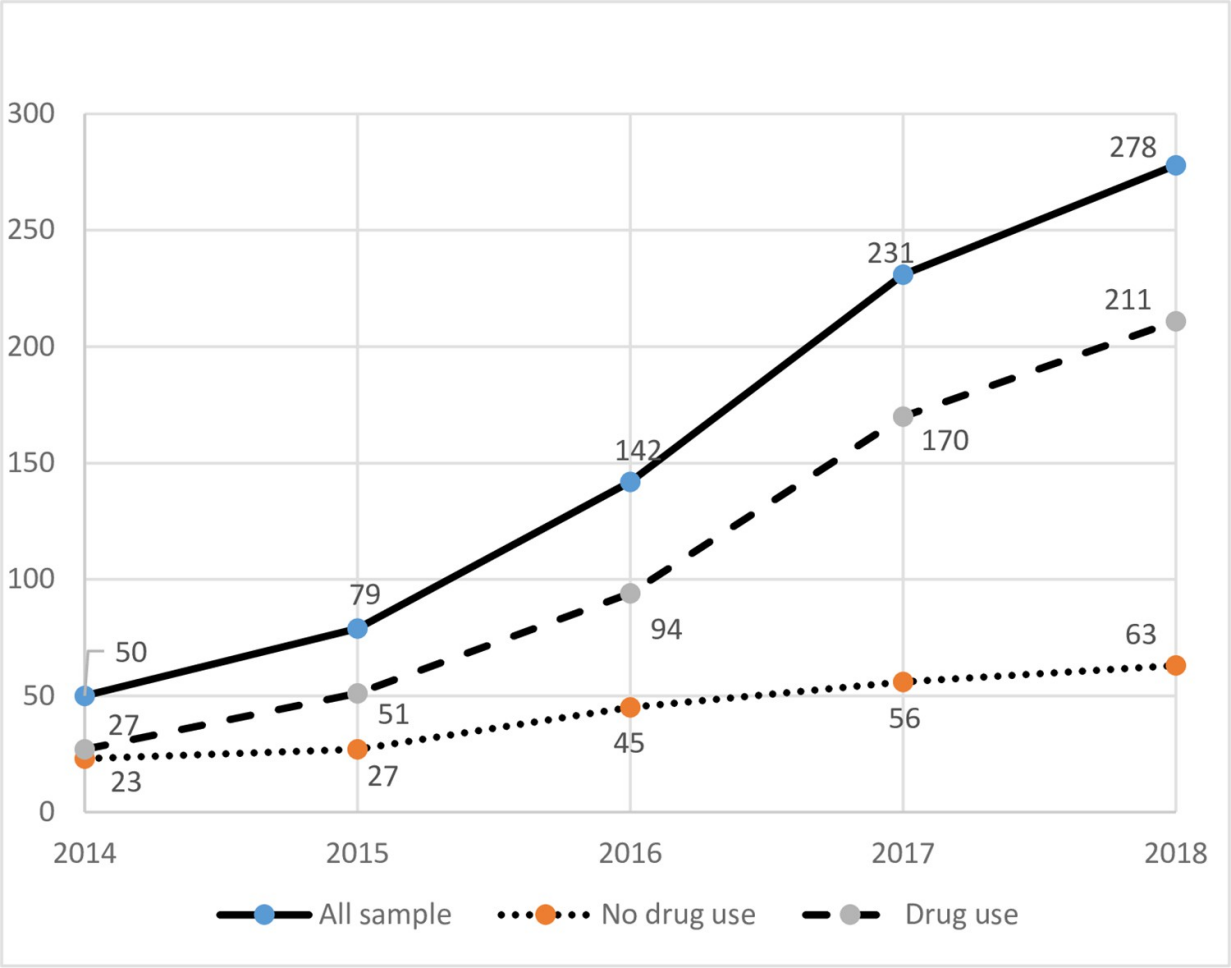
Frequency of infective endocarditis in West Virginia: 2014–2018.

A total of 780 patients with confirmed IE were admitted between January 1, 2014 and December 31, 2018 across the four study sites; the majority (63%) were admitted at one site, Ruby Hospital at West Virginia University. Table 1 depicts baseline characteristics of the overall sample stratified by drug use. The annual number of patients admitted with IE rose significantly over the study period, with two-thirds of the total admitted in the last two years. Most of this 458% increase occurred among patients with DU-IE (Fig 1); in fact, the proportion of DU-IE significantly increased by 681.5% over the study period, while non-DU-IE rose 173.9% (p = .004). Race was similar between groups, but the DU-IE group had more women (54.8% vs. 38.3%; p < .001). Patients with DU-IE were significantly younger, with a median age of 33.9 vs. 64.1 years (p < .001); the majority of DU-IE cases were in the 18–44 age group (83.2% vs. 12.2%, p < .001). Compared with patients with non-DU-IE, a majority of the patients with DU-IE reported being current smokers (78.8% vs. 26.6%; p < .001).

**Table 1:** Characteristics of patients with infective endocarditis.

	Total		No drug use		Drug use		p-value
	N = 780		N = 214	27.44%	N = 553	70.90%	
	N	%	N	%	N	%	
**Year**	** **						0.004
2014	50	6.41	23	10.75	27	4.88	
2015	79	10.13	27	12.62	51	9.22	
2016	142	18.21	45	21.03	94	17.00	
2017	231	29.62	56	26.17	170	30.74	
2018	278	35.64	63	29.44	211	38.16	
**Sex**							< 0.001
Female	390	50.0	82	38.32	303	54.79	
Male	390	50.0	132	61.68	250	45.21	
**Race**							0.173
White/Caucasian	736	94.36	202	94.39	523	94.58	
Other/Mixed	20	2.56	8	3.74	11	1.99	
Missing	24	3.08	4	1.87	19	3.44	
**Smoking status**							< 0.001
Current smoker	499	63.97	57	26.64	436	78.84	
Ex-smoker	112	14.36	66	30.84	43	7.78	
Non-smoker	98	12.56	70	32.71	26	4.70	
Missing	71	9.10	21	9.81	48	8.68	
**Alcohol use**							0.141
Current alcohol use	155	19.87	38	17.76	117	21.16	
Prior alcohol use	68	8.72	16	7.48	52	9.40	
No alcohol use	408	52.31	128	59.81	276	49.91	
Missing	149	19.10	32	14.95	108	19.53	
**Age**							< 0.001
18–44	496	63.59	26	12.15	460	83.18	
45–64	173	22.18	85	39.72	86	15.55	
65+	110	14.10	103	48.13	6	1.18	
**Age**	**Median**	IQR	**Median**	IQR	**Median**	IQR	** **
	38.15	23.15	64.09	19.91	33.88	12.97	< 0.001

Causative organisms and comorbidities presented in Table 2 demonstrate a significantly higher proportion of DU-IE patients with positive cultures for methicillin-resistant *Staphylococcus aureus* (MRSA) compared with non-DU-IE patients (46.8% vs. 29.9% of DU-IE; p < .001). Compared with patients with DU-IE, patients with non-DU-IE had significantly more co-morbid conditions, such as hypertension, type 2 diabetes, chronic lung disease, coronary artery disease, hyperlipidemia, chronic kidney disease, stroke, peripheral vascular disease, and cancer (p < .001). Psychiatric disorders were more prevalent among those with DU-IE compared with patients with non-DU-IE (51.2% vs. 17.3%; p < .001). These diagnoses did not solely reflect substance use disorder (SUD): 22.9% of patients with DU-IE were diagnosed with psychiatric disorders other than SUD. No patients with non-DU-IE had a diagnosed SUD, but 40.7% of those with DU-IE had an SUD. Psychiatric diagnoses, such as depression, anxiety, posttraumatic stress disorder, bipolar disorder, were common among those with SUD.

**Table 2:** Causative organisms and comorbidities among patients with infective endocarditis.

	Total		No drug use		Drug use		p-value
	N = 780		N = 214	27.44%	N = 553	70.90%	
	N	%	N	%	N	%	
**Gram positive bacteria**							
MRSA^a^	333	42.69	64	29.91	259	46.84	< 0.001
MSSA ^b^	203	26.03	48	22.43	154	27.85	0.127
Other Streptococci	69	8.85	29	13.55	39	7.05	0.005
Enterococci	60	7.69	27	12.62	32	5.79	0.001
Viridans Streptococci	26	3.33	10	4.67	16	2.89	0.222
**Gram negative bacteria**							
Serratia species	42	5.38	7	3.27	35	6.33	0.095
Klebsiella species	16	2.05	6	2.80	10	1.81	0.403
Escherichia coli	15	1.92	7	3.27	8	1.45	0.142
**Fungi**							
Candida species	48	6.15	12	5.61	36	6.51	0.644
**Negative cultures**	32	4.10	14	6.54	18	3.25	0.041
**Miscellaneous**							
Other	75	9.62	38	17.29	38	6.87	< 0.00
Polymicrobial	12	1.54	2	0.93	10	1.81	0.526
**Number of organisms**							0.039
1	618	79.23	158	73.83	449	81.19	
2 or more	120	15.38	39	18.22	80	14.47	
Missing	42	5.38	17	7.94	24	4.34	
**Comorbidities**							
Psychiatric disorders	320	41.03	37	17.29	283	51.18	< 0.001
Hypertension	230	29.49	140	65.42	90	16.27	< 0.001
Type 2 diabetes	123	15.77	84	39.25	391	7.05	< 0.001
Coronary artery disease	95	12.18	72	33.64	23	4.16	< 0.001
Chronic lung disease	89	11.41	45	21.03	43	7.78	< 0.001
Hyperlipidemia	85	10.90	66	30.84	19	3.44	< 0.001
Acute kidney injury	77	9.87	26	12.15	49	8.86	0.169
Chronic kidney disease	70	8.97	47	21.96	23	4.16	< 0.001
Stroke	61	7.82	29	13.55	32	5.79	< 0.001
Peripheral vascular disease	57	7.31	35	16.36	22	3.98	< 0.001
Metastatic infections	39	5.00	9	4.21	30	5.42	0.491
Cancer	37	4.74	28	13.08	9	1.63	< 0.001
**Number of comorbidities**							< 0.001
1	273	3500	36	16.82	236	42.68	
2	137	17.56	45	21.03	91	16.46	
3 or more	179	22.95	116	54.21	63	11.39	
0 or missing	191	24.49	17	7.94	163	29.48	
**Psychiatric disorders**	320	41.03	37	17.29	283	51.18	< 0.001
Substance use disorder	225	28.85	0	0.00	225	40.69	< 0.001
Depression	162	20.77	25	11.68	137	24.77	< 0.001
Anxiety	141	18.08	17	7.94	124	22.42	< 0.001
Bipolar disorder	41	5.26	1	0.47	40	7.23	< 0.001
PTSD^c^	36	4.62	0	0.00	36	6.51	< 0.001
Other	8	1.03	5	2.34	3	0.54	0.042
**Number of psychiatric disorders**							< 0.001
1	135	17.31	23	10.75	112	20.25	
2	89	11.41	12	5.61	77	13.92	
3 or more	937	11.92	1	0.47	92	15.64	
0 or missing	463	59.36	178	83.18	272	49.19	

^a^MRSA—*Staphylococcus aureus*, methicillin resistant

^b^MSSA—*Staphylococcus aureus*, methicillin sensitive

^c^PTSD—Post-traumatic stress disorder

Most patients with IE (70.9%) had used drugs before hospital admission (Table 3). Among them, almost all (96.9%) had injected drugs; with 86.3% who reported injecting opioids (e.g., fentanyl, carfentanil, heroin, codeine, morphine, and oxycodone); and 33.5% injecting amphetamines. Other drugs were also commonly used, including buprenorphine, cannabinoids, cocaine, and benzodiazepines; and 56.6% reported polysubstance use. EMR documentation indicated that 32.2% of patients with DU-IE received MOUD before hospital admission, and 27.9% received MOUD during hospitalization. Among those who received MOUD before hospitalization, 40% did not receive MOUD during hospitalization.

**Table 3:** Substance use characteristics among patients with drug-use associated infective endocarditis.

	N = 553	
	N	%
**Drug use method**		
Injection	536	96.93
Inhalation	209	37.79
Ingestion	101	18.26
**Type of drug**		
Opioids	477	86.26
Amphetamines	185	33.45
Cannabinoids	178	31.19
Buprenorphine	141	25.50
Cocaine	140	25.32
Benzodiazepines	93	16.82
Methadone	33	5.97
Ecstasy/MDMA	16	2.89
Other	16	2.89
**Number of drugs**		
1	214	38.70
2	101	18.26
3 or more	212	38.34
Missing	26	4.70
**MOUD** ^**a**^ **prior to hospital admission**		
Yes	178	32.19
No	224	40.51
Missing	151	27.31
**MOUD**^**a**^ **during hospitalization**		
Yes	154	27.85
No	394	71.25

^a^MOUD—Medication for Opioid Use Disorder

Patients had a median hospital stay of 20 days, IQR: 32 (Table 4). The median length of stay significantly differed between DU-IE and non-DU-IE (median: 25.5 vs. 15 days; p < .001). Discharge AMA was more common among DU-IE (20% vs. 1.4%; p < .001), as were readmissions (17.5% vs. 7%; p < .001). On the other hand, a significantly higher proportion of patients with non-DU-IE died while hospitalized (15.4% vs. 7.2% of DU-IE; p < .001). Overall, patients with IE received care and consultation from providers from multiple disciplines, most commonly: infectious diseases (96.3%), cardiac surgery (83%), cardiology (70.4%), and social work (49.6%). Almost all patients (94.6%) received multidisciplinary care involving three or more consultative services, and this phenomenon was similar between groups, except for psychiatric services/consultations (48.6% vs. 6.1%; p < .001).

**Table 4:** Hospital utilization by patients with infective endocarditis.

	Total		No drug use		Drug use		p-value
	N = 780		N = 214	27.44%	N = 553	70.90%	
	N	%	N	%	N	%	
**Discharge status**							< 0.001
Discharge alive	590	75.54	178	83.18	403	72.88	
Discharge AMA^a^	115	14.85	3	1.40	110	19.89	
Death	75	9.60	33	15.42	40	7.23	
**Discharge location**							< 0.001
Home	521	66.71	113	52.80	397	71.79	
Skilled/other nursing facility	56	7.17	41	19.16	15	2.71	
Another hospital/facility	52	6.66	12	5.61	40	7.23	
Residential substance use treatment facility	33	4.35	0	0.00	33	5.97	
Acute rehab	17	2.18	11	5.14	6	1.08	
Long-term acute care facility	16	2.05	4	1.87	12	2.17	
Missing	10	1.28	0	0.00	10	1.81	
**Surgery**							< 0.001
Yes	295	37.77	57	26.64	237	42.86	
No	484	62.10	157	73.36	315	56.96	
**Medical consults**							
Infectious Disease	751	96.28	209	97.66	532	96.20	0.316
Cardiology	549	70.38	168	76.50	370	66.91	0.002
Nephrology	268	34.36	94	43.93	170	30.74	< 0.001
Dentistry	189	24.23	37	17.29	152	27.49	0.003
Neurology	130	16.67	57	26.64	73	13.20	< 0.001
Vascular	114	14.62	45	21.03	69	12.48	0.003
Ophthalmology	71	9.10	19	8.88	52	9.40	0.822
**Surgical consults**							
Cardiac Surgery	648	83.08	161	75.23	477	86.26	<0.001
General Surgery	135	17.31	41	19.16	93	16.79	0.458
Orthopedic Surgery	104	13.33	22	10.28	81	14.62	0.125
Neurosurgery	75	9.62	21	9.81	53	9.57	0.892
Interventional Radiology	75	9.62	19	8.88	53	9.57	0.89
**Other consults**							
Social work	387	49.62	105	49.07	282	50.99	0.632
Physical/Occupational therapy	320	41.03	114	53.27	206	37.25	< 0.001
Psychiatry	283	36.28	13	6.07	269	48.64	< 0.001
Spiritual counseling	176	22.56	49	22.90	126	22.78	0.974
Pain management	75	9.62	1	0.47	74	13.38	< 0.001
Individual therapy	18	2.31	1	0.47	17	3.07	0.032
**Number of consultations**							0.090
1	16	2.05	1	0.47	13	2.35	
2	22	2.82	6	2.80	16	2.89	
3	168	21.54	39	18.22	123	23.24	
4	149	19.10	47	21.96	99	17.90	
5	81	10.38	30	14.02	50	9.04	
6	66	8.46	22	10.28	44	7.96	
7 or more	274	35.13	68	31.78	206	37.25	
Missing	4	0.51	1	0.47	2	0.36	
**Readmission**							< 0.001
Yes	112	14.36	15	7.01	98	17.69	
No	668	85.64	199	92.99	456	82.31	
**Length of stay**							< 0.001
4 days or less	76	9.74	14	6.54	57	10.31	
5–9 days	113	14.49	48	22.43	64	11.57	
10–19 days	190	24.36	77	35.98	109	19.71	
20–29 days	121	15.51	45	21.03	75	13.56	
30–39 days	62	7.95	7	3.27	54	9.76	
40–49 days	120	15.38	11	5.14	108	19.53	
> = 50 days	94	12.05	11	5.14	83	15.01	
Missing	4	0.51	1	0.47	3	0.54	
	**Median**	IQR	**Median**	IQR	**Median**	IQR	
**Length of Stay**	20	32	15	15	25.5	34	< 0.001

^a^AMA—Against Medical Advice

In the multivariable regression analysis controlling for sex, age, number of comorbidities, and surgery, patients with DU-IE were three times as likely to be discharged AMA/have in-hospital mortality compared to patients with non-DU-IE (OR: 2.99; 95% CI: 1.67–5.64), driven by the high AMA discharge rate for this group (Table 5). Sex, age, and surgery were also significant predictors of discharge AMA/in-hospital mortality. Females and patients ≥65 years were at higher odds of discharge AMA/in-hospital mortality while patients who underwent surgery were at lower odds of discharge AMA/in-hospital mortality. However, the number of comorbidities was not a significant predictor of discharge against medical advice/in-hospital mortality.

**Table 5:** Multivariable logistic regression: Discharge against medical advice/in-hospital mortality vs. discharge alive.

Variable	Unadjusted OR	95% CI	Adjusted OR	95% CI
Drug use (Y/N)	1.840	(1.240, 2.790)	2.985	(1.673, 5.640)
Sex (M/F)	1.658	(1.192, 2.316)	1.496	(1.060, 2.117)
Age (18-<65, 65–90)	0.953	(0.584, 1.512)	2.202	(1.114, 4.460)
Comorbidities (0, 1–2, 3+)	0.825	(0.649, 1.046)	0.974	(0.738, 1.287)
Surgery (Y/N)	0.468	(0.322, 0.671)	0.442	(0.300, 0.641)

## Discussion

In this retrospective study, we document a striking 458% increase in overall IE admissions in West Virginia over a five-year period ending in 2018, largely attributable to the 681% increase in DU-IE. Findings also show drug use was an independent predictor of discharge against medical advice/in-hospital mortality. There was a significant burden of care in patients with DU-IE who more commonly underwent cardiac surgery and received psychiatric services/consultations compared with patients with non-DU-IE. Multidisciplinary care involving three or more consultative services was common across the entire study population reflecting the serious nature of an IE diagnosis.

This study supports other recent reports of increases in IE cases, particularly among people who inject drugs [5–7, 14]. While the increase in DU-IE cases was steep, there was also a gradual increase in the non-DU-IE cases. Findings from our study show that patients with non-DU-IE were significantly older (median age 64 years), and over half had three of more comorbidities such as type 2 Diabetes and coronary artery disease. Previous studies have also demonstrated an overall increasing incidence of IE, including among the non-drug-use associated patients [5, 15].

However, our IE population had a higher proportion of DU-IE cases (71%) than that reported for other regions of the U.S. Data from the U.S. National Inpatient Sample (NIS) indicated that DU-IE cases accounted for up to 23.5% of IE cases during January 2010 –August 2015 [16], while a report from North Carolina indicated that DU-IE cases accounted for only 11% of IE cases during 2007–2017 [5]. The high proportion of DU-IE cases in our sample may be attributable to both the completeness of our data from manual chart review rather than relying only on ICD codes, and the disproportionately high prevalence of injection opioid use in WV. Indeed, WV is considered the epicenter of the opioid crisis, having three times the U.S. average rate of fatal opioid overdoses [17].

The younger median age of patients with DU-IE compared to non-DU-IE patients in our sample aligns with several other studies [15, 18–22]; few studies have reported a higher average age among the DU-IE population [6, 23]. The prolonged length of stay [14, 24] for patients with DU-IE is also similar to that described by other studies. This finding likely reflects the unique challenges associated with DU-IE, the need for continued hospitalization to complete a course of intravenous antimicrobials, operative complexity, and SUD treatment to avoid recidivism and early mortality [25].

Our results show a significantly higher proportion of MRSA IE cases among the DU-IE patients. The epidemiology of *Staphylococcus aureus* has changed in the US in recent years. CDC data show that injection drug use-associated MRSA infections have more than doubled from 4.1% in 2012 to 9.2% in 2016 [26]. MRSA infections are over 16 times more likely to develop in persons who inject drugs [26]. Data from the neighboring state of Tennessee that shares similar demographic, social, and public health characteristics with WV, also show a significant 17.7% increase in MRSA infections during 2015–2017, with almost a quarter related to IDU [27].

That patients with DU-IE were approximately half as likely to die during their hospitalization than those with non-DU-IE may be attributable to their considerably younger age and significantly fewer cormorbid medical conditions. Readmission of patients with DU-IE was more than double that for non-DU-IE, which may be related to having been discharged against medical advice and complications due to recrudescent injection drug use. Moreover, the readmission rate for DU-IE is likely an underestimate since patients may have been admitted to a local hospital without cardiac surgery availability and not transferred to a study hospital.

Our study identified drug-use as an independent risk-factor for patients who were discharged AMA or who died during hospitalization, after controlling for other potential confounders. This aligns with an analysis from the representative National Inpatient Sample of over 30,000 IE hospitalizations between 2010 and 2015 that also found injection drug use to be significantly associated with increased odds of discharge AMA [16].Our study also found that 40% of patients who had received MOUD before admission were not prescribed MOUD during hospitalization. This represents a missed opportunity to provide continued SUD care that may reduce the risk of recidivism [28].

Due to its retrospective design, our study was limited by the lack of availability of baseline variables such as education, income, duration of all drug use and specifically of injection drug use. Errors in diagnostic coding may have introduced misclassification of some patients. In addition, patients may not have consistently reported, or the EMR may not have consistently captured, relevant information on drug, alcohol, and cigarette use, and some DU-IE cases may have been misclassified. We also found that three quarters of patients receiving MOUD during hospitalization had an SUD diagnosis, which suggests underreporting of SUD. Finally, we lack long-term follow-up data on patient outcomes such as out of hospital mortality or subsequent admissions to other hospitals.

Despite these potential limitations, a major strength of our study is that rather than relying solely on ICD codes as multiple prior studies have done [6, 9, 20, 29, 30], we validated IE and drug use diagnoses with other clinical indicators through a detailed chart review. Use of ICD code combinations for identification of DU-IE are not specific and frequently fail to identify DU-IE cases correctly [31]. Manual chart review conducted in our study provided granular clinical information, and thereby, improved the accuracy of inclusion of IE cases and identification of drug use.

Additionally, we examined individual patients, and not hospitalizations based on discharge databases, as was done in other studies. By focusing on the four academic hospitals, three of which are the only hospitals in WV that offer cardiac surgery, our study included the largest number of IE patients who are routinely transferred to hospitals where state-of-the-art care can be provided. Thus, our database captured most of the IE cases in WV during the five-year study period, which is close to comprising a state registry of all IE cases in this period.

## Conclusions

In summary, robust statewide EMR data reveal a 456% increase in overall IE admissions over five years in WV, primarily attributable to increases in DU–IE. Compared with patients with non-DU-IE, those with DU-IE exhibited a significant burden of psychiatric conditions, in addition to SUD. Despite lower in-hospital mortality, individuals with DU-IE had significantly higher resource utilization and required not only usual multidisciplinary care, but also cardiac surgery and MOUD. These data provide a crucial foundation to focus on preventive harm reduction efforts and to develop strategies for specialized treatment of DU-IE to improve outcomes, limit the proportion who are discharged against medical advice, and prevent readmissions.
